# Prevalence and risk factors of superior segmental optic hypoplasia in a Korean population: the Korea National Health and Nutrition Examination Survey

**DOI:** 10.1186/1471-2415-14-157

**Published:** 2014-12-15

**Authors:** Sam Seo, Chong Eun Lee, Dai Woo Kim, Young Kook Kim, Jin Wook Jeoung, Chan Yun Kim, Se Woong Kang, Ki Ho Park, Epidemiologic Survey Committee of the Korean Ophthalmological Society

**Affiliations:** Department of Ophthalmology, Seoul National University Hospital, Seoul National University College of Medicine, 101 Daehak-roJongno-gu, Seoul, 110-744 Korea; Department of Ophthalmology, Jeju National University Hospital, 15, Aran-13-gil, Jeju-si, Jeju, 690-767 Korea; The Institute of Vision Research, Department of Ophthalmology, Yonsei University College of Medicine, 50 Yonsei-ro, Seodaemun-gu, Seoul, Korea; Department of Ophthalmology, Samsung Medical Center, Sungkyunkwan University School of Medicine, 81 Irwon-ro, Gangnam-gu, Seoul, Korea

**Keywords:** Superior segmental optic hypoplasia, Prevalence, Risk factor, Korea National Health and Nutrition Examination survey

## Abstract

**Background:**

This study investigated the prevalence and risk factors for superior segmental optic hypoplasia (SSOH) in a Korean population based on the data from the nationwide Korea National Health and Nutrition Examination Survey (KNHANES).

**Methods:**

We performed a retrospective review of the KNHANES dataset covering January 2012 to December 2012. The study population comprised 5,612 subjects (≥19 years of age) who had participated in a medical interview covering demographic and systemic information, been issued a questionnaire regarding associated SSOH risk factors including gender, age, systemic disease, and family history, and had undergone an ophthalmologic examination. Two masked readers evaluated fundus photography, paying special attention to the presence of SSOH. Associations of risk factors (identified in the medical interview portion) with SSOH prevalence were investigated using multivariate logistic regression analysis.

**Results:**

SSOH was detected in 16 eyes of 14 subjects, or 0.24% of the 5,612 subjects. All 16 eyes showed a corresponding visual-field defect. In multivariate logistic regression analyses, maternal history of diabetes (Odds ratio (OR), 7.666; 95% Confidence interval (CI), 2.601 ~ 22.593, p < 0.001) and paternal history of ischemic heart disease (IHD) (OR, 11.105; CI, 3.361 ~ 36.686, p < 0.001) were associated with increased risk of SSOH.

**Conclusions:**

This study provides the first representative population-based data on SSOH prevalence in Korea. Additionally, multivariate analyses revealed that a history of maternal diabetes and paternal IHD was the most important factor influencing the prevalence of SSOH.

## Background

Superior segmental optic hypoplasia (SSOH) is a non-progressive congenital optic nerve anomaly characterized by a relative superior entrance of the central retinal artery, pallor of the superior optic disc, superior peripapillary scleral halo, and thinning of the superior retinal nerve fiber layer (RNFL) [1]. Patients with SSOH typically have good visual acuity and an inferiorly located visual-field defect in the affected eyes [2].

Petersen & Walton described a specific form of optic nerve hypoplasia characterized by a relative hypoplasia of the superior portion of the optic nerve head [3]. The term superior segmental optic hypoplasia, or SSOH, became generally accepted after Kim et al.’s investigation of this condition’s association with maternal type I diabetes mellitus [1]. The pathogenesis of this condition, however, remains unclear. Although association with maternal diabetes has been strongly suggested, cases occurring in the absence of maternal diabetes also been reported [2, 3].

Yamamoto et al. recently reported a Japanese SSOH prevalence rate of 0.3% [4]. On the basis of this relatively high prevalence, they emphasized the need for greater awareness in clinical practice. In this light, we considered it prudent to undertake a study on the prevalence and risk factors for SSOH in Korea.

In the present study, we determined the prevalence of SSOH using data from the Korean National Health and Nutrition Examination Survey (KNHANES), a large population-based, cross-sectional survey. Additionally, we investigated the ocular and systemic parameters to identify the risk factors associated with SSOH.

## Methods

### Sample and population

The data used in the present study were obtained from KNHANES 2012, which covers the months from January to December of that year. The KNHANES a cross-sectional and nationally representative survey that was conducted by the Ministry of Health and Welfare of the Republic of Korea and the Korean Ophthalmological Society. Using a multistage, stratified, probability-clustered sampling method and a weighting scheme, the detailed survey design can produce estimated health statistics representative of the civilian, non-institutionalized Korean population. This study has followed the Tenets of the Declaration of Helsinki. As the data of KNHANES is opened to the public after removal of personal identifiers and being anonymized (http://knhanes.cdc.go.kr), the Institutional Review Board of Seoul National University Hospital determined that this study was exempt from requiring their approval. This study included KNHANES data on 5,612 individuals aged 19 years or older.

### Survey components

All of the participants underwent a personal medical interview and an ophthalmological examination as part of the KNHANES process. The interview covered demographic and systemic information. For assessment of overall general medical condition, including any history of ocular disease, systemic disease, family history and current medication, a standardized questionnaire was administered.

Participants were asked whether any biological member of their family, living or decreased, had ever been told he/she had diabetes, hypertension, or ischemic heart disease (IHD). We defined family history as having a first-degree relative (parent) with diabetes, hypertension, or IHD diagnosed by a doctor and treated with medication, regardless of disease severity or duration. Family history was categorized as maternal and paternal.

The full ophthalmologic examination included logMAR visual acuity measurements, auto-refraction, slit-lamp examination for anterior segment of the eye, intraocular pressure (IOP) measurements, and fundus photography. Refraction measurements were converted into spherical equivalents and calculated as the spherical value plus half the astigmatic value. IOP was measured with a Goldmann applanation tonometer (Haag-Streit, Inc., Bern, Switzerland). Digital fundus images were taken with a digital non-mydriatic fundus camera (TRC-NW6S; Topcon, Inc., Tokyo, Japan).

Visual-field testing was performed with frequency-doubling technology (FDT) (Humphrey Matrix; Carl Zeiss Meditec, Inc., Dublin, CA) using the N-30-1 screening test. The test location was deemed abnormal if not identified on two attempts at a contrast level identified by 99% of the healthy population.

Two independent readers who were masked to all information other than the fundus photographs, evaluated the color fundus photography for the presence of abnormalities of the optic nerve head and RNFL. Discrepancies between the two observers’ findings were resolved by consensus. In case of no consensus the final decision was made by the third reader, the principal investigator. In this study, SSOH was defined as rim thinning of the optic nerve head in the superior or superonasal region with corresponding RNFL defects in at least one eye [4].

### Statistical analysis

Fisher’s exact test was utilized to compare the demographic characteristics. A multivariate logistic regression models were applied to the determination of the SSOH risk factors, adjusting for potential confounders. Adjusted means, 95% CIs, and 2 sided P values were obtained. Statistical significance was defined as a P value less than or equal to 0.05.

## Results

During the 12-month KNHANES period, 5,612 participants aged 19 years or older underwent fundus photography. In the present study, fundus photographs of 11,087 eyes of those 5,612 cases were successfully reviewed. 137 eyes was excluded due to media opacities or other difficulties such as small pupil or poor focus. The age and gender distributions of the study participants are shown in Table 1. Optic disc anomalies were detected in 53 eyes of 48 subjects with a prevalence rate of 0.48% per eye and 0.86% per subject. Optic disc drusen were detected on the fundus photographs of 10 eyes of nine patients. Myelinated nerve fibers were detected in 27 eyes of 24 subjects. Of the 5,612 cases reviewed, we found 16 eyes of 14 cases with SSOH (about 0.14% of the eyes, and about 0.24% of the cases). All of the 16 eyes showed corresponding inferior visual-field defect. SSOH affected both eyes in two cases and one eye in 12 cases. The clinical features of the SSOH participants are provided in Table 2. The visual acuity in the SSOH group was 0.07 ± 0.10, and in the normal group, 0.17 ± 0.33. The worst visual acuity in the SSOH group was 0.3. The SSOH group showed lower logMAR scores (better visual acuity) than were recorded for the normal group. This finding is consistent with a previous study, which showed that patients with SSOH typically have good visual acuity. The mean refraction in the SSOH group was −1.06 ± 1.86 D, which was similar to that in the normal group (−0.80 ± 2.17 D).

**Table 1:** **Participants by age and gender**

Age (years)	Men	Women	Total
19-29	258	381	639
30-39	383	555	938
40-49	397	550	947
50-59	444	632	1076
60-69	433	580	1013
70-79	362	469	831
≥80	57	111	168
Total	2334	3278	5612

**Table 2:** **Clinical data on superior segmental optic hypoplasia (SSOH) cases reviewed in present study**

No.	Age (years)	Laterality	BCVA (logMAR)	IOP	Refraction (SE; Diopters)
1	19	OS	0	15	−3.75
2	24	OD	0	15	−1.50
3-1	29	OD	0.1	16	2.25
3-2	29	OS	0.1	16	1.75
4	34	OD	0.2	13	−3.25
5	38	OD	0	15	−3.75
6	38	OD	0	14	0.25
7	41	OD	0.1	18	−1.25
8-1	42	OD	0	13	−0.25
8-2	42	OS	0	13	−0.50
9	44	OS	0	10	−0.63
10	49	OS	0	11	−1.50
11	59	OS	0	16	2.25
12	63	OD	0.2	19	−0.56
13	64	OD	0.1	17	−4.38
14	65	OS	0.3	13	−2.63

BCVA = Best corrected visual acuity; logMAR = Logarithm of the Minimum Angle of Resolution; SE = Spherical equivalent; IOP = Intraocular pressure.

The results of the ocular and systemic parameter comparison are summarized in Table 3. The participants’ ages ranged from 19 to 65 years (mean ± SD: 43.50 ± 14.89 years). The visual acuity was better than or equal to 20/20 in eight (57.14%) and worse than 20/25 in three (21.43%) of the 14 eyes with SSOH. When the randomized one eye was selected in bilateral cases and the affected eyes were selected in unilateral cases, the refractive error as spherical equivalent was −1.06 ± 1.86 D (mean ± SD; range: −4.38 to 2.25 D); the IOP was 14.68 ± 2.60 mmHg (mean ± SD; range: 10 to 19 mmHg). There was no statistically significant difference between SSOH and the controls in age, gender, history of hypertension, diabetes or IHD.

**Table 3:** **Parameter differences between SSOH and healthy subjects**

	SSOH subjects (n = 14 Subjects)	Healthy subjects (n = 5,598 Subjects)	*P*value ^*^
**Ocular**			
Refractive error (SE, diopter)	−1.06 ± 1.86	−0.80 ± 2.17	0.261
Intraocular pressure (mmHg)	14.68 ± 2.60	13.78 ± 2.68	0.192
**Systemic**			
Age (years)	43.50 ± 14.89	51.55 ± 16.81	0.079
Gender (female)	8/14	3270/5598	0.923
Systemic hypertension	2/14	1366/5598	0.379
Ischemic heart disease	1/14	192/5598	0.446
Diabetes	0/14	488/5598	0.248

SSOH = superior segmental optic hypoplasia; SE = Spherical equivalent.

The numerical values are shown as the mean ± standard deviation (with the range in parentheses).

The values of refractive error and intraocular pressure (IOP) were randomly selected for both eyes.

^*^P value based on Mann–Whitney U test, P < 0.05 significant.

A Fisher’s exact test revealed that a paternal history of hypertension and IHD and a maternal history of diabetes were significant risk factors for SSOH (Table 4). In the final logistic regression model for SSOH risk, the independent factors that remained significant were paternal history of IHD and maternal history of diabetes (Table 5). Paternal history of IHD (OR, 11.105; 95% CI, 3.361 ~ 36.686) and maternal history of diabetes (OR, 7.666; 95% CI, 2.601 ~ 22.593) were both associated with increased risk of SSOH. Paternal history of hypertension did not significantly affect predisposition to SSOH.

**Table 4:** **Associations between SSOH prevalence and systemic factors by cross-tables**

Factor	SSOH subjects (n = 14 Subjects)	Healthy subjects (n = 5,598 Subjects)	Odds ratio (95% CI)	P-value ^*^
**Systemic**				
HTN	2/14	1366/5598	0.516 (0.115-2.310)	0.539
IHD	1/14	192/5598	2.166 (0.282-16.641)	0.388
Diabetes	0/14	488/5598		0.627
**Family history**				
Paternal HTN	5/14	853/5598	3.090 (1.033 - 9.244)	**0.05**
Maternal HTN	4/14	1113/5598	1.612 (0.505 - 5.149)	0.498
Paternal IHD	4/14	155/5598	14.046 (4.358 - 45.279)	**<0.001**
Maternal IHD	1/14	150/5598	2.794 (0.363 - 21.495)	0.318
Paternal diabetes	1/14	450/5598	0.880 (0.115 - 6.742)	1.0
Maternal diabetes	6/14	428/5598	9.060 (3.129 - 26.230)	**<0.001**

SSOH = superior segmental optic hypoplasia; 95% CI = 95% confidence interval.

HTN = systemic hypertension; IHD = ischemic heart disease.

*P-values based on Fisher’s exact test; P < 0.05 significant.

Significant values are shown in bold.

**Table 5:** **Logistic regression analysis of SSOH-associated factors**

Factor	Odds ratio	95% CI	*P*value ^*^
Paternal history of HTN	2.249	0.726 - 6.961	0.160
Paternal history of IHD	11.105	3.361 - 36.686	**<0.001**
Maternal history of diabetes	7.666	2.601 - 22.593	**<0.001**

SSOH = superior segmental optic hypoplasia; 95% CI = 95% confidence interval.

HTN = systemic hypertension; IHD = ischemic heart disease.

*P-values based on logistic regression; P < 0.05 significant.

Significant values are shown in bold.

## Discussion

This study analyzed the data from a national health survey (KNHANES) and determined the prevalence and risk factors for SSOH. SSOH prevalences have been reported in a number of Asian studies. Yamamoto et al. reported that prevalence of SSOH in Japan, as determined by fundus photography, was 0.3% among their subjects and 0.2% among the general population [4]. Han et al., reviewing photographs of 3,905 hospital-based Korean subjects, calculated a 0.08% prevalence [5]. However, those study subjects were not representative of the general Korean population but only of the one institute where they were examined.

Our study demonstrated that the prevalence of SSOH in Korea is actually as high as 0.24%. The discrepancy in the prevalence of SSOH between the present study and Han’s study is attributable to the difference in the study population. Our study participants constituted a representative Korean population, while those in Han’s study are patients who had visited a health promotion center in a tertiary referral hospital. Han et al. noted, as a limitation, the possibility that participants with a known optic disc anomaly might not have been included. Additionally, different examinations were used for SSOH diagnosis. A combination of stereo disc photography, red-free retinal nerve fiber layer, and Humphrey visual field test was used in the study by Han et al., whereas fundus photography and frequency-doubling technology (FDT) perimetry were used in our study. The difference in the diagnostic ability between these examination sets would have led to the difference in the prevalence.

In fact, ours is the first study to investigate the Korean prevalence of SSOH in a representative random population. Considering the ethnic similarity between Koreans and Japanese along with Yamamoto et al.’s result, we believe that the prevalence of SSOH obtained in our study is an accurate estimate of its true prevalence.

SSOH has been noted to occur both unilaterally and bilaterally with relatively equal frequency [2, 6]. The percentage of unilateral SSOH in our series was slightly higher than those reported by other investigators. There were no statistically significant differences in prevalence by age or gender. Although several reports have indicated a female predisposition to SSOH [1, 3, 7], no significant gender difference has yet been documented anywhere. Further research will be necessary for helpful clarification of such issues.

Tendencies toward lower birth weight, shorter gestation time and poorer control of maternal diabetes were associated with increased risk of SSOH [8]. These associations could be due to unfavorable conditions during development that affect fetal growth, general fetal development and the risk of malformation. Brodsky et al. suggested, based on cases of identical twins, genetic susceptibility as a possible risk of SSOH [6]. Unoki et al. also noted a familial case of SSOH [9]. In any case, the pathogenic mechanism of SSOH remains obscure.

The results of this study indicated that maternal history of diabetes and paternal history of IHD are significantly associated with SSOH. Whereas reported risk factors for SSOH have been relatively rare in the literature, several studies have shown a correlation between insulin-dependent diabetic mothers and SSOH in their children [7, 8]. Our present study served to reconfirm the positive relationship between maternal history of diabetes and SSOH in children. A possible pathology is selective interference of the teratologic mechanism of insulin-dependent diabetes mellitus with the early-gestational development of superior retinal ganglion cells.

As for paternal history of IHD, it has gone completely unreported in the literature, and remains to be confirmed in future studies. Our present results, uniquely, showed paternal history of IHD to be an independent risk factor for SSOH. A possible explanation is related to altered levels of growth factors and insulin sensitivity. Several studies have reported an association between optic nerve hypoplasia and endocrine disturbances including growth hormone (GH) deficiency and insulin resistance [10, 11]. Paternal IHD has been reported for offspring with insulin resistance [12], which association could increase the risk of optic nerve hypoplasia.

Paternal IHD was found to be associated with the GH level of offspring. Previously, paternal IHD has been reported for offspring with polymorphism in angiotensin converting enzyme (ACE) [13]. ACE variation, moreover, has been associated with lower levels of growth hormone and insulin-like growth factors, because the ACE polymorphism site is located nearby the GH gene site [14]. It is not clear if the finding of the association with the GH level of offspring, then, would explain an increased risk of SSOH. However, this association is a possible explanation of genetic susceptibility to SSOH. Because determinative genetic information could not be obtained in this study, and because the association between paternal IHD and congenital malformation remains controversial, further study in efforts to prove that association certainly is warranted.

This study has several limitations. The analysis relied on self-reported data on medical conditions. As a result, the prevalences could have been underestimated or influenced by the individual’s memory. Another limitation of the current analysis is that we were unable to assess disease onset, duration, or severity, as pertinent data were not collected by KNHANES.

Notwithstanding the limitations of the present study, the strength of this study is that the sample size is significantly large, and indeed, KNHANES is based on a representative sample of the Korean population. Therefore, the proportions and odds ratios obtained in this study are representative of the Korean population as well.

## Conclusions

We determined that the prevalence of SSOH in Korea is 0.24%. To our knowledge, this is the first study on SSOH prevalence based on data representing a Korean population (KNHANES). We conclude that individuals with a history of maternal diabetes and paternal IHD might be at a higher risk of SSOH.

## Abbreviations

SSOH: Superior segmental optic hypoplasia
KNHANES: Korea National Health and Nutrition Examination Survey
OR: Odds ratio
CI: Confidence interval
IHD: Ischemic heart disease
RNFL: Retinal nerve fiber layer
IOP: Intraocular pressure
FDT: Frequency doubling technology.
